# Management of Sepsis in Asplenic Patients

**Published:** 2013-05-06

**Authors:** Piazza Ornella

**Affiliations:** Department of Medicine and Surgery, University of Salerno, Salerno, Italy

## EDITORIAL VIEW

Overwhelming post-splenectomy infection (OPSI) is a severe disease that can progress from flu-like disturbances to fulminant sepsis since patients who are asplenic are at an increased risk for infection and death from microorganisms. To prevent OPSI, several measures are essential and must begin before a scheduled surgery or just after an urgent splenectomy. One proposed prevention measure is to enhance partial surgery when it is possible [ 1 ] . Although the introduction of an adequate vaccination programme against incapsulated bacteria and oral penicillin prophylaxis have decreased the overall risk of OPSI, its mortality rate is about 50–70% within 48 hours from onset.

Considering that OPSI can develop in a very short time period and that its related mortality rate worsens with delayed or inadequate treatment, it is important for Haematologists, Anaesthesiologists and Emergency Physicians to be familiar with it. In this number of Translational Medicine, Serio and Co-workers [ 2 ] present a 12-year survey describing the management of this relatively rare disease. The Authors cleverly refer to the importance of an aggressive and properly targeted early treatment: according to dr Morgan and Tomich [ 3 ] intensive care manoeuvres, including empiric antibiotics, and early goal-directed therapy, are the keys to successful treatment and if initiated early in the patient’s course, the 70% mortality rate can be significantly reduced.

Dr Serio et Al considerations about OPSI may be cautiously enlarged to the population of patients affected by malignancies of the lymphatic system or other tumours, who underwent chemotherapy. Infections in patients under immunosuppression, additionally enhanced by chemotherapy, often lead to the development of severe sepsis, associated with high mortality. Severe sepsis is currently defined as the presence of SIRS (systemic inflammatory response syndrome), a source of infection, and dysfunction of at least one vital organ [ 4 ] . Appropriate treatment includes detection and elimination of the source of infection as well as supporting organ function by maintaining tissue perfusion. State of the art therapy is summarized in the recently updated Surviving Sepsis Campaign (SSC) guidelines [ 5 ] . The clinical goals of the evidence-based bundled strategies include optimizing timeliness in the delivery of care and creating a continuum for sepsis management that runs from the emergency department to the acute and critical care settings.

The course of sepsis is rapid. Patient outcomes improve when sepsis is diagnosed and treated quickly. OPSI and all the infection in hematologic malignancies patients are devastating: implementing the SSC guideline through an education programme is feasible and may result in early therapy (within 6 hours) with aggressive fluid administration and appropriate antibiotics, with relevant positive, impact on the outcome.

I wish that a Sepsis Education Programme may be implemented in every hospital, starting from teaching hospitals where early and correct therapeutic interventions will contribute to the survival benefits of OPSI patients.
